# Incorporation of membrane-bound, mammalian-derived immunomodulatory proteins into influenza whole virus vaccines boosts immunogenicity and protection against lethal challenge

**DOI:** 10.1186/1743-422X-6-42

**Published:** 2009-04-24

**Authors:** Andrew S Herbert, Lynn Heffron, Roy Sundick, Paul C Roberts

**Affiliations:** 1 Center for Molecular Medicine and Infectious Diseases, Department of Biomedical Sciences and Pathobiology, Virginia Maryland Regional College of Veterinary Medicine, Virginia Tech, 1981 Kraft Drive, Blacksburg, VA 24060, USA; 2 Department of Immunology/Microbiology, Wayne State University School of Medicine, 7374 Scott Hall, 540 E. Canfield Ave., Detroit, MI 48201, USA

## Abstract

**Background:**

Influenza epidemics continue to cause morbidity and mortality within the human population despite widespread vaccination efforts. This, along with the ominous threat of an avian influenza pandemic (H5N1), demonstrates the need for a much improved, more sophisticated influenza vaccine. We have developed an in vitro model system for producing a membrane-bound Cytokine-bearing Influenza Vaccine (CYT-IVAC). Numerous cytokines are involved in directing both innate and adaptive immunity and it is our goal to utilize the properties of individual cytokines and other immunomodulatory proteins to create a more immunogenic vaccine.

**Results:**

We have evaluated the immunogenicity of inactivated cytokine-bearing influenza vaccines using a mouse model of lethal influenza virus challenge. CYT-IVACs were produced by stably transfecting MDCK cell lines with mouse-derived cytokines (GM-CSF, IL-2 and IL-4) fused to the membrane-anchoring domain of the viral hemagglutinin. Influenza virus replication in these cell lines resulted in the uptake of the bioactive membrane-bound cytokines during virus budding and release. *In vivo *efficacy studies revealed that a single low dose of IL-2 or IL-4-bearing CYT-IVAC is superior at providing protection against lethal influenza challenge in a mouse model and provides a more balanced Th_1_/Th_2 _humoral immune response, similar to live virus infections.

**Conclusion:**

We have validated the protective efficacy of CYT-IVACs in a mammalian model of influenza virus infection. This technology has broad applications in current influenza virus vaccine development and may prove particularly useful in boosting immune responses in the elderly, where current vaccines are minimally effective.

## Background

Influenza epidemics continue to cause morbidity and mortality within the human population. Yearly epidemics affect 5–20% of the population leading to over 200,000 hospitalizations and up to 36,000 deaths annually in the United States [1]. The economic impact of influenza related illness costs the United States upwards of $167 billion dollars per year [1]. The recent emergence of highly pathogenic avian influenza (HPAI) H5N1 has significantly raised awareness and concern of a pending pandemic flu event. Prior to 1997, it was thought that HPAI circulating in avian species could not be directly transmitted to humans. However, recent studies have documented that HPAI can cross the avian-human species barrier and infect humans, leading to disease and high mortality (50%) [2-4]. Furthermore, recent incidences of low-grade human-to-human transmission of H5N1 have heightened concerns that an H5N1 pandemic may occur [5]. Continual yearly outbreaks of influenza and the looming threat of a potential influenza pandemic illustrate the growing need for improved influenza vaccines.

The ability of adjuvants to enhance vaccine efficacy have been well documented, yet the current commercially available influenza vaccines in the United States do not utilize any licensed form of adjuvant. Oil adjuvants, such as incomplete Freund's adjuvant, have long been known to boost the immune response to co-administered antigens; however these oil-based adjuvants are not ideal adjuvant candidates due to potential side effects [6]. Recent studies have begun to look at other methods of boosting the immune response to influenza antigens using adjuvants such as alum, MF59, and Quil A, as well as Influenza-Immunostimulating Complex (ISCOM), an immune complex comprised of influenza antigen, cholesterol, lipid, and saponins [7-10].

Immunomodulatory proteins such as cytokines and chemokines have been evaluated for their ability to augment vaccine immunogenicity in numerous vaccine candidates. Cytokines and chemokines such as RANTES, IL-12, IL-6, and GM-CSF, delivered as either soluble protein or plasmid expression vector, have proven to boost the immune responses to co-administered antigens [11-13]. While the adjuvant potential of cytokines and chemokines are clearly demonstrated in these studies, two major problems arise for those vaccines using soluble forms of cytokines and chemokines, (1) dispersion of the protein from the site of administration and (2) the short half-life of the protein. It has been suggested that immunomodulators may function better if they are maintained in close proximity or juxtaposed to antigens and remain in their bioactive state for a longer period of time [14-17]. Recently, encapsulation or fusion of immunomodulators (GM-CSF, IL-2) directly to the cognate antigen has been shown to significantly augment immune responses [18-21]. Clearly, presentation of immunomodulators in close association with antigen greatly increases the immunogenicity of the antigen.

As a means to boost the immunogenicity of whole virus vaccines or even subunit vaccines, we postulated that inactivated virus particles bearing membrane-bound immunostimmulatory molecules would elicit a more robust and balanced humoral immune response to influenza virus. Here, we describe studies demonstrating the ability of CYT-IVACs (cytokine bearing influenza virus vaccines) to boost antiviral humoral immune responses and protect against lethal challenge using a mouse model of infection.

## Methods

### Construction of expression plasmids

Mouse-derived granulocyte macrophage-colony stimulating factor (mGM-CSF) and interleukin 2 and 4 (mIL-2, mIL-4) were fused to a short stalk, transmembrane, and cytoplasmic tail domain of influenza A/WSN/33 hemagglutinin (HA) using standard PCR methodologies as described previously [22]. Briefly, primers, amplifying the carboxyl terminal 71 amino acids of WSN HA and the coding sequence of the cytokines, were designed to introduce the appropriate restriction sites. Nucleotides 1521–1730 coding for the 26 amino acid stalk region, the transmembrane domain, and cytoplasmic tail domain of the hemagglutinin were amplified using the forward primer 5'-CCGGATCCAATGGGACTTATGATTATCC-3' and the reverse primer 5'-CCGAATTCTCAGATGCATATTCTGCACTGC-3' to introduce restriction sites Bam HI and Eco RI (underlined), respectively. Primers specific for mGM-CSF (forward 5'-CCAAGCTTGGAGGATGTGGCTGCAGAA-3'; reverse 5'-GGGGATCCTTTTTGGACTGGTTTTTTGC-3'), mIL-2 (forward 5'-CCGGTACCAGCATGCAGCTCGCATCCTGTGTC-3'; reverse 5'-GGGGATCCTTGAGGGCTTGTTGAGATGA-3'), and mIL-4 (forward 5'-CCGGTACCGCACCATGGGTCTCAACCCCCA-3'; reverse 5'-CCGGATCCCGAGTAATCCATTTGCATGATG-3') were designed to remove stop codons and introduce Hind III (mGM-CSF) or Kpn I (mIL-2 and mIL-4) and BamHI endonuclease restriction sites on the 5' and 3' ends respectively. PCR products were generated using Platinum *Pfx *(Invitrogen) and GeneAmp PCR System 2400 (Applied Biosystems). Purified PCR products were subsequently digested and inserted into the respective restriction sites of pcDNA3.1 using T4 DNA Ligase (Invitrogen) according to the manufacturers protocol. Plasmid constructs, harboring the respective fusion constructs, were sequenced by the Wayne State University Sequencing Core (Applied Genomics Technology Center) to verify sequence and integrity of the constructs.

### Generation of CYT-IVAC producer cell lines

Madin-Darby canine kidney (MDCK) cells were maintained in complete growth media (DMEM/10% FBS) consisting of Dulbecco's Modified Eagles Media supplemented with 10% fetal bovine serum (Atlanta Biologicals) and the antibiotics penicillin/streptomycin (100 U/100 μg). Cells were transfected with expression plasmids using Lipofectamine2000 (Invitrogen) as described previously [22]. Stable transfectants were selected by growth in DMEM/10%FBS supplemented with Geneticin (1.5 mg/ml; Gibco). Geneticin-resistant cells were subcloned by limiting dilution plating in 96-well plates in the presence of Geneticin (G418™ Invitrogen, 1 mg/ml). Individual MDCK subclones were screened for cell surface expression and bioactivity of the respective membrane-bound cytokines.

### Viral infection, purification, and inactivation

Wild-type and CYT-IVAC producer MDCK cells (90% confluent) were infected at an MOI of 1 with either influenza virus A/PR/8/34 (H1N1) or A/Udorn/72 (H3N2). Following virus adsorption (1 hr, 37°C), the inoculum was removed and DMEM/2% FBS was added. Supernatants from infected monolayers were harvested 24–36 hours post infection and cellular debris was pre-cleared at *400 × g *for 15 minutes at 4°C. Virions were purified by centrifugation through two sequential 10–26% iodixanol continuous gradients (OptiPrep™, Axis-Schield) (SW41 rotor, *55,000 × g*, 45 min at 4°C). Banded virus was collected and concentrated by centrifugation at *88,000 × g *for 45 minutes at 4°C and subsequently re-suspended in phosphate-buffered saline, PBS. Purified virus was inactivated by treatment with 15 mM β-propiolactone for 15 minutes at 25°C. The reaction was neutralized by the addition of sodium thiosulfate (40 mM final concentration, 30 min, 25°C). Inactivated virus was diluted with PBS, pelleted by centrifugation as described and resuspended in sterile PBS. Total viral protein concentration was determined using a bicinchoninic acid protein assay kit (Pierce Biotechnology). Inactivation was confirmed by monitoring cytopathic effect in MDCK cells treated with 5 μg of inactivated virus vaccine for a period of 3–5 days at 37°C in the presence of 1.5 μg/ml TPCK-treated trypsin (Sigma).

### Cell surface expression and viral incorporation of membrane-bound cytokines (Immunofluorescence Microscopy)

MDCK cells were grown to 90% confluency on glass cover slips in 24 well plates. Cells were washed with phosphate buffered saline (PBS) and fixed with 3% paraformaldehyde (PF) in 250 mM HEPES for 10 minutes at room temperature (RT). PF was removed and 50 mM glycine in PBS was added for 10 minutes at RT to quench any remaining PF. Cells were washed 2 times with PBS and blocked with 2% chicken serum in PBS for 30 minutes at RT. For immunostaining cells were incubated sequentially with rat anti-cytokine specific antibody (BD Pharmagen) and chicken anti-rat IgG conjugated Alexa Fluor^® ^488 antibody (Invitrogen/Molecular Probes). All antibodies were diluted in PBS/2% chicken serum. Cover slips were mounted on slides using ProLong Antifade (Invitrogen/Molecular Probes). Immunofluorescent staining was visualized using a Nikon E800 Epifluorescence Microscope. Digital images were captured using a Roper CoolSnap FX digital camera and analyzed using MetaMorph Imaging Software (Universal Imaging).

To visualize viral incorporation of membrane-bound cytokines, CYT-IVAC producer cells, grown on cover slips, were infected with filamentous influenza A/Udorn/72 at an MOI of 1. The cells were fixed at 8 hr post-infection with 3% PF and blocked as described above. Cells were incubated with rat anti-cytokine specific primary antibody and Alexa Fluor^® ^488 conjugated secondary antibody as described above. Additionally, cells were incubated with goat anti-H3 antibody and secondary chicken Alexa Fluor^® ^594 conjugated anti-goat IgG (Invitrogen/Molecular Probes). Cover slips were mounted and immunofluorescence was analyzed as described above.

### Western blot analysis

Vaccines were solubilized in Laemmli Buffer (BioRad) (LB) and heated at 96°C for 10 minutes to denature proteins. Samples were separated on 12% PAGE-SDS and subsequently blotted to PVDF membrane. Membranes were probed by sequential incubation with rat anti-GM-CSF (BD Bioscience), followed by goat anti-rat IgG horseradish-peroxidase conjugated secondary antibody (Santa Cruz). Membranes were exposed to ECL or Femto solution per manufacturers (Pierce) instructions and membranes were visualized using Chemdoc XRS (BioRad).

### Total Cytokine and Hemagglutinin Quantitation by Slot Blot Assay

Serial dilutions of vaccines at 1, 0.5 and 0.25 μg (cytokine quantification) or 1, 0.2 and 0.04 μg (HA quantification) of total viral protein, as well as serial diluted recombinant cytokine (2000 ng to 1.95 ng) were blotted on PVDF membranes using a slot blot apparatus. Membranes were blocked with 5% milk solution and subsequently incubated sequentially with diluted primary antibody, specific for the respective cytokine (rat anti-GM-CSF, IL-2, or IL-4, BD Bioscience) or hemagglutinin (mouse anti-HA, Meridian Life Science^® ^Inc or rabbit anti-H1N1/Pan H1, Pierce^® ^Inc) followed by the respective horseradish-peroxidase conjugated secondary antibody (goat anti-rat IgG (Santa Cruz), goat anti-mouse IgG (BioRad) or goat anti-rabbit IgG (Sigma). Membranes were exposed to ECL or Femto solution per manufacturers (Pierce^®^) instructions and chemiluminescent signals were recorded using a Chemdoc XRS (BioRad). Images were processed with ImageJ software (NIH freeware) and standard curves for each cytokine were generated using optical pixel densities. Total cytokine content for each vaccine preparation was extrapolated from standard curves and is expressed as the average of the three dilutions evaluated for each vaccine in nanograms (ng) of cytokine per microgram (μg) of total viral protein. The signal intensity of the HA specific signal for each vaccine was calculated for each dilution and the average pixel density per μg of total viral protein is given.

### Hemagglutination Assay

Hemagglutination units (HAU) were determined by agglutination of chicken red blood cells as previously described [23]. Briefly, serial diluted vaccine preparations were mixed with an equal volume of fresh 0.5% chicken red blood cells and incubated at room temperature for 30 minutes. Red blood cell agglutination was recorded and HAU per μg of total viral protein is expressed as the reciprocal of the last dilution of virus that resulted in agglutination.

### Bioassays of membrane-bound cytokines

Bone marrow (BM) cells, as indicator cells for mGM-CSF bioactivity, were prepared from the femurs of female Balb/c mice. Briefly, bone marrow was flushed from the femurs with RPMI and the cell suspension passed through a 70 μm cell strainer. Red blood cells were lysed using RBC lysis buffer (155 mM NH_4_Cl, 10 mM KHCO_3_, 0.01% EDTA). Cells were washed 2 times with RPMI and re-suspended in complete RPMI (10% FBS, 20 mM L-glutamine, 1 M HEPES, 100 mM Sodium Pyruvate, 55 μM 2β-Mercaptoethanol, Penicillin/Streptomycin (100 units/100 μg/ml)). For MDCK based bioassays, BM cells (2 × 10^5^/well) were added to wells of a 96 well plate containing 90% confluent, mitomycin C (50 μg/ml) treated wild type or CYT-IVAC producer (mGM-CSF~HA) MDCK cells. For virus based bioassays and quantitation of viral incorporated bioactive GM-CSF, BM cells (2 × 10^5^) or MPRO cells (5 × 10^3^) [24], respectively, were added to wells of a 96 well plate containing inactivated A/PR/8/34 wild type or A/PR/8/34 mGM-CSF~HA. Recombinant GM-CSF was also used to establish a standard curve by which virus-incorporated bioactive GM-CSF could be quantitated. Plates were incubated at 37°C for 72 hours (BM) or 48 hours (MPRO cells). For the last 18 hours of incubation for the cell-based bioassay, cells were pulsed with ^3^H-thymidine then harvested and counted using a scintillation counter. For the viral based bioassay, Alamar Blue^® ^(Invitrogen) was added to each well at 10% of the total volume for the last 24 hours and Alamar Blue^® ^reduction was determined from the absorbance values recorded at 570 nm and 600 nm after 72 (BM) or 48 (MPRO) hours.

CTLL-2 cells (a gift from Dr. Robert Swanborg, Wayne State University) were used as indicator cells for the bioactivity of mIL-2. Cells were maintained in complete RPMI supplemented with recombinant mouse IL-2 (10 ng/ml). CTLL-2 cells (5 × 10^3^) were added to 96 well plates containing mitomycin C treated cells (wild-type or mIL-2 CYT-IVAC producer cells) or inactivated virus (A/PR/8/34 wild-type or A/PR/8/34 mIL-2~HA) as described above. Recombinant IL-2 was also used to establish a standard curve by which virus-incorporated bioactive IL-2 could be quantitated. Plates were incubated at 37°C for 48 hours. For the last 18 hours of incubation for the cell-based bioassay, cells were pulsed with ^3^H-thymidine then harvested and counted using a scintillation counter. For the virus particle based bioassay, Alamar Blue^® ^was added to each well for the last 24 hours and absorbance was read at 570 nm and 600 nm after 48 hours.

CT.4s cells (gift from Dr. William Paul and Dr. Jane Hu-Li, Laboratory of Immunology, National Institute of Health) were used to determine mIL-4 bioactivity [25]. Cells were maintained in complete RPMI supplemented with recombinant mouse IL-4 (2 ng/ml). CT.4s cells (5 × 10^3^) were added to 96 well plates containing mitomycin C treated MDCK cells (wild-type or mIL-4 CYT-IVAC producer cells) or inactivated virus (A/PR/8/34 wild-type or A/PR/8/34 mIL-4~HA) as described above. Recombinant IL-4 was also used to establish a standard curve by which virus-incorporated bioactive IL-4 could be quantitated. Plates were incubated at 37°C for 48 hours. For the last 18 hours of incubation for the cell-based bioassay, cells were pulsed with ^3^H-thymidine, harvested and counted using a scintillation counter. For the viral based bioassay, Alamar Blue^® ^was added to each well for the last 24 hours and absorbance was read at 570 nm and 600 nm after 48 hours.

Standard curves for recombinant GM-CSF, IL-2 and IL-4 were deduced from the difference data of the 570 nm and 600 nm absorbance readings for each dilution of recombinant protein using Prism (GraphPad Software, Inc.). Difference data, collected from various dilutions of GM-CSF, IL-2, or IL-4-bearing CYT-IVAC preparations, was applied to their respective standard curve for quantitation of bioactive membrane-bound cytokine for each CYT-IVAC on a per microgram basis.

### Vaccination studies

Animal experiments were performed in accordance with NIH guidelines and with approval by the Institutional Animal Care and Use Committee of the Virginia State University and Polytechnic Institute. Groups of 8–10 week old female Balb/c mice (NCI, Charles, River Laboratories) were immunized subcutaneously with 0.375 μg total viral protein of β-propiolactone inactivated A/PR/8/34 wild-type, A/PR/8/34 mGM-CSF~HA, A/PR/8/34 mIL-2~HA, or A/PR/8/34 IL-4~HA diluted in PBS. PBS alone acted as the negative vehicle control. Serum was collected on day 21 post-vaccination by retro-orbital bleeding. Mice were challenged with 1000 TCID_50 _of mouse-adapted Influenza A/PR/8/34 (100 LD_50_) on day 35 post-vaccination. Weight loss and survival was monitored following challenge.

### Enzyme linked immunosorbent assay (ELISA)

Antiviral antibody levels in sera of vaccinated animals were determined by a standard enzyme-linked immunosorbent assay using whole virus as the coating antigen. Briefly, Immuno Plates (Nunc) were coated with 10 hemagglutination units (HAU) of inactivated A/PR/8/34 in coating buffer (sodium bicarbonate, pH 9.6) and blocked overnight at 4°C in PBST buffer (phosphate buffered saline with 0.05% Tween 20) supplemented with 2% BSA. Plates were washed 3 times with wash buffer (PBS containing 0.05% Tween 20). Serum samples, collected on day 21 post vaccination, were added to wells of ELISA plates and plates were incubated with shaking overnight at 4°C. Plates were washed 3 times with PBST. Horseradish Peroxidase (HRP) conjugated secondary antibody (anti-mouse IgG, IgG_1_, or IgG_2a_; Southern Biotech), diluted in PBST with 2% BSA, was added and plates were incubated with shaking for 1.5 hours at RT. Plates were washed 3 times with wash buffer and wells were incubated with substrate (2,2'-Azino-Bis(3-Ethylbenzthiazoline-6-Sulfonic Acid; Sigma) for 30 minutes at RT, followed by the addition of 1% SDS to stop the reaction. Absorbance was measured at 405 nm using a plate reader (SpectraFluor Plus, Tecan) and O.D. readings were plotted against a standard curve to determine the amount of influenza specific antibody per milliliter of serum.

### Microneutralization Assay for determination of virus neutralizing antibody titers

Neutralizing antibody titers were determined for serum samples collected from mice on day 21 post-vaccination as described in the WHO Manual on Animal Influenza Diagnosis and Surveillance [26]. Briefly, two-fold serial dilutions of serum in PBS were incubated with 100 TCID_50 _of influenza A/PR/8/34 for 1 hour at room temperature. The serum/virus cocktail was added to MDCK cells for 1 hour at 37°C. Serum/virus cocktail was removed and cells were incubated for 3 days at 37°C in the presence of 1.5 μg/ml TPCK-treated trypsin (Sigma). Neutralizing titer was determined to be the reciprocal of the last dilution of serum that protected MDCK cells from cytopathic effect.

### Quantitation of viral loads in lungs

Viral loads in the lung tissue of vaccinated mice were determined by collecting lungs on day 4 post-challenge. Lungs were weighed and flash frozen in DMEM with liquid nitrogen. Lung tissue was homogenized, pelleted and supernatants were collected. Lung homogenates were brought to equal volume with DMEM. Viral titers of lung homogenates were determined from serial 10-fold sample dilutions and incubation with MDCK cells for 1 hour at 37°C to allow for virus adsorption. Subsequently, cells were washed and incubated for 3 days at 37°C in the presence of 1.5 μg/ml TPCK-treated trypsin (Sigma) and cytopathic effects were recorded. Viral loads were reported as 50% tissue culture infectious dose units (TCID_50_/ml) as determined by the Reed-Muench method [27].

### Statistics

Statistical analysis using Prism software (Graphpad) was conducted with the help of Dr. Stephen Were (statistician for VA-MD Regional College of Veterinary Medicine). ELISA antibody titer data was analyzed by One-way ANOVA on normalized log transformed data using Dunnett's multiple comparison test with PR/8/34 wild-type group as the control. Comparison of survival curves was analyzed using Fisher's exact test.

## Results

### Establishment of CYT-IVAC producer cell lines for the production of Cytokine-Bearing Influenza Vaccines (CYT-IVACs)

We have previously described an *in vitro *cell culture platform that allows for the direct incorporation of membrane-bound forms of chicken-derived cytokines into virus particles [22]. Preparation of these cytokine-bearing influenza virus vaccines, or CYT-IVACs, requires that the cytokine or immunomodulator of choice be both anchored in the virion membrane, and efficiently packaged into virions as they are released from the infected host cell. Further, the membrane-bound immunomodulator must retain its bioactivity. To ensure successful membrane anchoring and virion packaging, a gene encoding for full-length cytokine (including its signal sequence) is fused inframe to a gene segment encoding a short extracellular stalk domain, the transmembrane spanning and the cytoplasmic tail domains of the influenza virus hemagglutinin. Alternatively, genes encoding mature soluble forms of cytokines or chemokines can be fused inframe to the N-terminal encoding cytoplasmic tail, membrane-spanning and short stalk domains of the viral neuraminidase [22].

**Figure 1:**
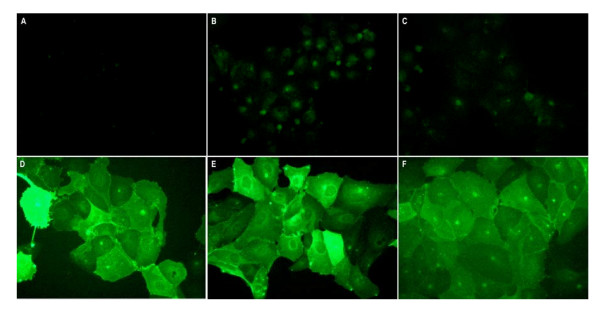
**Cell surface expression of membrane-bound immunomodulator fusion constructs**. Cell surface immunofluorescent staining of wild-type MDCK cells (A, B, C) and MDCK CYT-IVAC producer cells expressing membrane-bound mouse GM-CSF/HA (D), IL-2/HA (E), or IL-4/HA (F). Paraformaldehyde fixed cells were labeled using rat anti-GM-CSF (A, D), anti-IL2 (B, E) or anti-IL4 (C, F) specific antibodies followed by Alexa Flour^® ^488 conjugated secondary antibody.

In the present study, mouse derived IL-2, IL-4 and GM-CSF were fused inframe to the C-terminal portion of the hemagglutinin and inserted into the mammalian expression vector pcDNA3.1 (Invitrogen) under control of the CMV promoter element; pcDNA3.1~mIL-2/HA, ~mIL-4/HA and ~mGM-CSF/HA respectively. Following establishment of stable MDCK transfectants expressing the membrane-bound cytokines, cell surface expression was confirmed by immunofluorescence microscopy using cytokine-specific antibodies. As depicted in Figure 1, cell surface expression of GM-CSF/HA, IL-2/HA or IL-4/HA could be readily demonstrated in MDCK cells stably transfected with the respective expression constructs (Figure. 1D, E, and 1F respectively). Positive staining was absent in vector control MDCK transfected cells using each the cytokine specific antibodies (Figure. 1A, B, and 1C). Stable MDCK transfectants were subcloned by limiting dilution to ensure maximal surface expression of the fusion constructs and further selected based upon i) cell surface expression of the membrane-bound cytokines, and ii) cell surface bioactivity of the specific membrane-bound cytokines as further described below.

**Figure 2:**
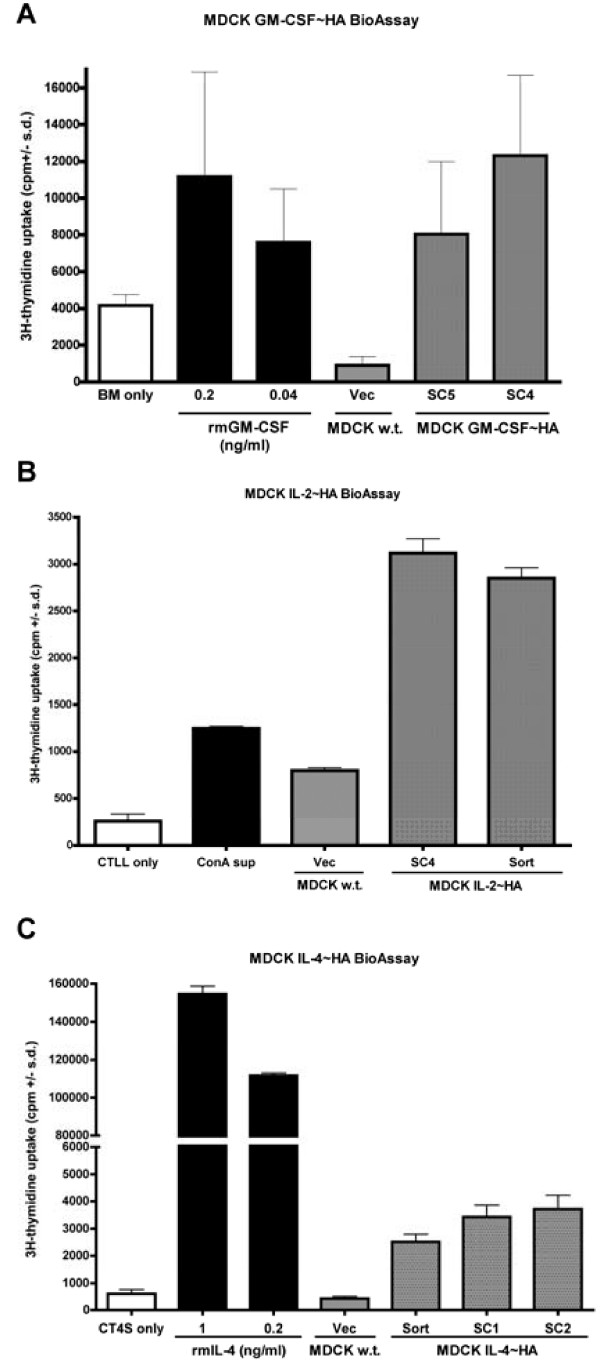
**Membrane-bound immune-modulators are bioactive on the surface of MDCK CYT-IVAC producer cells**. Mitomycin C treated subclones (SC) or FACS sorted (sort) CYT-IVAC producer cells expressing murine GM-CSF (A), IL-2 (B), or IL-4 (C) or wild-type MDCK cells were co-cultured with cytokine specific indicator cells, bone marrow (BM), CTTL-2 and CT.4s respectively. Proliferation of cytokine responsive cell lines was measured by ^3^H-thymidine incorporation. Recombinant protein was used as positive control.

Membrane-bound cytokine bioactivity was determined using specific cell-based bioassays in which MDCK transfectants, wild-type or subclones of membrane-bound cytokine producing cells, were incubated with cytokine specific indicator cells (Figure 2). Bioactivity or proliferation was based on the incorporation of ^3^H-thymidine. All three stably transfected MDCK cell lines expressing either mGM-CSF/HA, mIL-2/HA, or mIL-4/HA induced the proliferation of their respective indicator cell line at levels well above background (indicator cells alone). Vector control or wild-type MDCK cells failed to induce significant proliferation of indicator cell lines. These results confirm that the mGM-CSF, mIL-2, and mIL-4 fusion constructs are expressed in a bioactive form on the cell surface of our CYT-IVAC producer cells.

### Viral incorporation of membrane-bound cytokines

**Figure 3:**
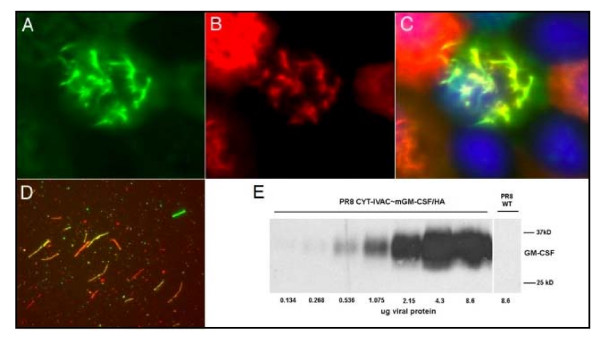
**Membrane-bound immunomodulators are incorporated during budding and release of virions from influenza virus infected cells**. MDCK CYT-IVAC producer cells infected with filamentous influenza virus A/Udorn/72 were stained at 8 hr post-infection with antibodies specific for mGM-CSF (A, green) and hemagglutinin (B, red). Images A and B are overlaid to depict co-localization of mGM-CSF and full-length HA to budding viral filaments (C). Released virus particles collected from supernatants of infected CYT-IVAC producer cells stained for GM-CSF and HA as described above (D). Western blot of gradient purified virus derived from GM-CSF/HA expressing MDCK cells or wild-type MDCK cells (E) and probed for the presence of GM-CSF.

Our goal in this study was to produce inactivated whole virus vaccines, which exhibit immunopotentiating capacity compared to standard, unadjuvanted influenza whole virus vaccine. In order for membrane-bound cytokines to serve as immunopotentiating adjuvants they must first be packaged efficiently into budding virions, and subsequently retain their bioactivity following inactivation of the virus particles. To confirm packaging of membrane-bound cytokines into virions, we initially took advantage of our work with filamentous strains of influenza virus [28-30]. Filamentous strains allow for visualization of virus particles budding from infected cells or of virions released into the extracellular media using indirect immunofluorescence microscopy techniques. To assess whether membrane-bound cytokines at the surface of MDCK cells were incorporated into budding virions, stable MDCK transfectants were infected with filamentous influenza A/Udorn/72 (H3N2) virus and at 8 hours post-infection, fixed and immunostained with antibodies specific for the respective cytokines or for the viral hemagglutinin glycoprotein (HA). As demonstrated in Figure 3 (A–D), budding filamentous virions clearly incorporated membrane-bound GM-CSF when propagated in infected MDCK~GM-CSF/HA expressing cells. Co-localization (yellow fluorescence) was evident indicating that both membrane-bound GM-CSF and full-length, virally encoded HA were incorporated into budding viral filaments. Importantly, localization of GM-CSF/HA and full length HA was also confirmed on virions collected from the supernatants of infected producer cells (Figure 3D).

To further confirm cytokine incorporation into virions, virus harvested from infected producer cells was gradient-purified and inactivated with β-propiolactone. Complete virus inactivation was confirmed using a tissue culture infectious dose assay, which monitors virus induced cytopathicity or production of hemagglutinating virus particles. None of the inactivated CYT-IVACs (5 μg of purified virus) resulted in the production of hemagglutinating virus particles or cytopathic effect in wild-type MDCK cells over a 5 day monitoring period. Western blot analysis and slot blot assays were performed on gradient purified CYT-IVACs to further verify cytokine incorporation and to quantitate the total amount of virus-incorporated cytokine, respectively. In addition, the HA content of gradient purified wild-type and CYT-IVAC vaccine preparations was evaluated using slot blot and hemagglutination assays to rule out any potential adverse effects on packaging of full-length viral HA. As depicted in Figure 3E using western blot analysis, the presence of mGM-CSF/HA was detected only in progeny virions harvested from A/PR/8/34 infected mGM-CSF/HA producer MDCK cells and not in virions collected from A/PR/8/34 infected wild-type MDCK cells. GM-CSF was detectable in as little as 0.268 μg of total viral protein. Using standard curves derived from slot blots of recombinant GM-CSF, IL-2 or IL-4, we were further able to quantitate the amount of virus-incorporated cytokine for each CYT-IVAC (Table 1). The GM-CSF and IL-4-bearing CYT-IVACs incorporated relatively high levels of membrane-bound cytokines, 185 ng GM-CSF and 176 ng IL-4 per μg of vaccine respectively, compared to the IL-2-bearing CYT-IVAC, only 4.924 ng IL-2 per μg of vaccine. Due to lack of a suitable HA standard for A/PR/8/34 hemagglutinin, we were unable to precisely quantitate the viral HA content. However, we were able to compare the relative HA amounts based on optical density scans of western or slot blot assays in which equal amounts of purified viral protein were loaded. Using this approach, the HA content across vaccine preparations did not differ significantly when equal amounts of viral protein were probed with either monoclonal or polyclonal antibodies specific for H1 hemagglutinin (Table 1). Additionally, hemagglutination units per μg of viral protein for wild-type and CYT-IVAC vaccines did not differ significantly, indicating comparable relative full-length HA content for wild-type and CYT-IVAC vaccines (Table 1).

**Table 1:** Characterization of CYT-IVAC hemagglutinin and cytokine content

**Vaccine**	HA pixel density*	**HAU/μg of vaccine**	**Total cytokine ** (ng/μg vaccine)**	**Bioactive cytokine ** (pg/μg vaccine)***
**PR/8/34 w.t**.	5835.4	16	NA	NA
**PR/8/34 GM-CSF/HA**	6407.9	16	185 ± 21	87.3
**PR/8/34 IL-2/HA**	5562.9	32	4.92 ± 0.3	411
**PR/8/34 IL-4/HA**	6090.4	32	176 ± 24	456

* Pixel density of HA specific chemiluminescent signal following equal loading of total viral protein

** Quantitation of virus-incorporated cytokine on protein level based on standard curve of recombinant cytokine (ng of cytokine per ug of vaccine)

*** Quantitation of virus-incorporated cytokine on bioactive level based on standard curve of recombinant cytokine (pg of cytokine per ug of vaccine)

In these latter studies, influenza virus A/PR/8/34, a spherical particle-producing virus, was used to prepare vaccines. Thus, incorporation of membrane-bound cytokine is neither restricted to a morphological phenotype nor a particular influenza virus subtype. Additional studies in our laboratory have further confirmed membrane-bound cytokine incorporation using H6N2 avian strains of influenza virus for the infection (data not shown).

### Bioacitivty of membrane-bound cytokines following viral inactivation

**Figure 4:**
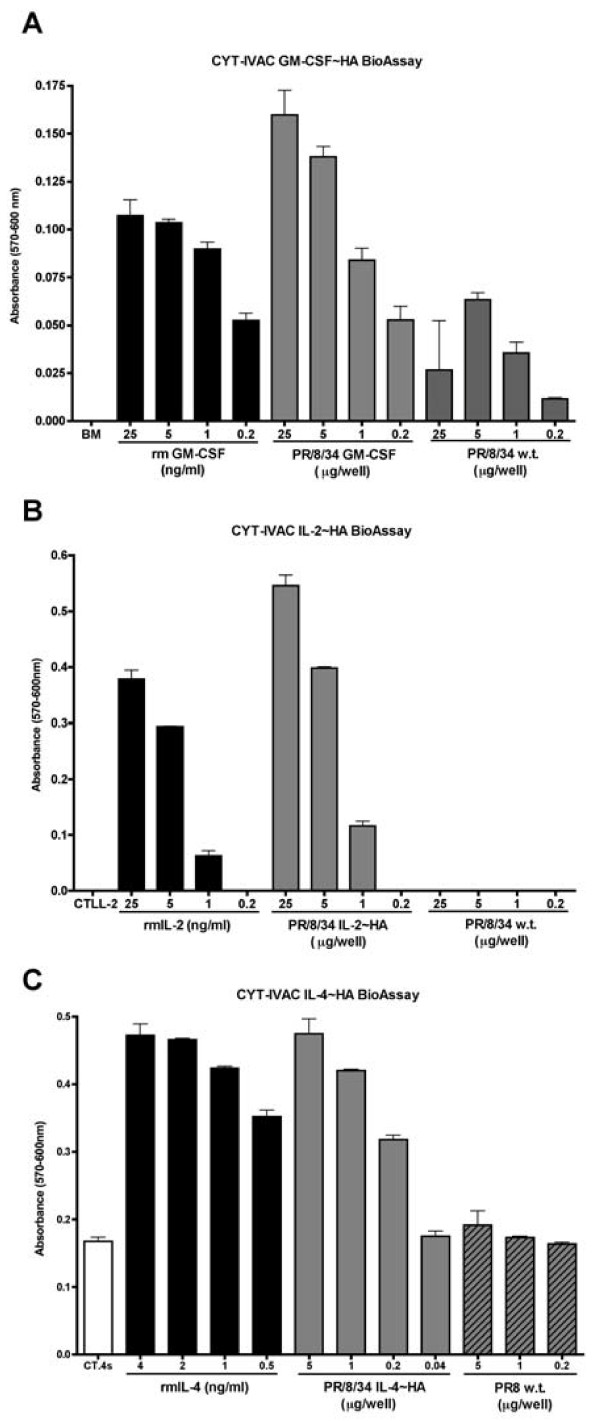
**Membrane-bound immunomodulators retain bioactivity following viral inactivation**. Cytokine specific indicator cell lines (bone marrow cells, BM; CTTL-2; or CT.4s) were incubated with decreasing concentrations of β-propiolactone inactivated wild-type vaccine or GM-CSF CYT-IVAC (A), IL-2 CYT-IVAC (B) or IL-4 CYT-IVAC (C). Proliferation was determined by Alamar Blue^® ^reduction. Recombinant protein was used as the positive control.

Inactivated, gradient purified CYT-IVACs were subsequently analyzed by bioassay using the appropriate indicator cells. Wild-type inactivated virus harvested from vector control MDCK cells was used as a negative control and proliferation was monitored by either ^3^H-thymidine incorporation or reduction of Alamar Blue^®^. Alamar Blue^® ^is a safe, non-radioactive alternative to^3^H-thymidine and it has been proven to be as sensitive and reproducible, in proliferation assays, as^3^H-thymidine [31]. As depicted in Figure 4, CYT-IVACs bearing mGM-CSF/HA, mIL-2/HA, and mIL-4/HA, all retained their bioactivity following β-propiolactone inactivation inducing significant proliferation of their respective indicator cell lines compared to wild-type inactivated virus. In addition to the above-mentioned quantitation of virus-incorporated cytokine by slot blot assays, we thought it necessary to quantitate the biologically active membrane-bound cytokine to better indicate the dose of cytokine delivered during vaccination. Despite the relatively low level of virus-incorporated IL-2 compared to IL-4, the amount of biologically active IL-2 and IL-4 present in the respective CYT-IVACs was comparable at 0.411 ng IL-2 and 0.456 ng IL-4 perμg of vaccine, respectively (Table 1). In contrast, the amount of bioactive membrane-bound GM-CSF for the GM-CSF CYT-IVAC was considerably lower (87.3 pg perμg of vaccine) despite the relatively high level of virus-incorporated GM-CSF as determined by the slot blot assay (Table 1).

**Figure 5:**
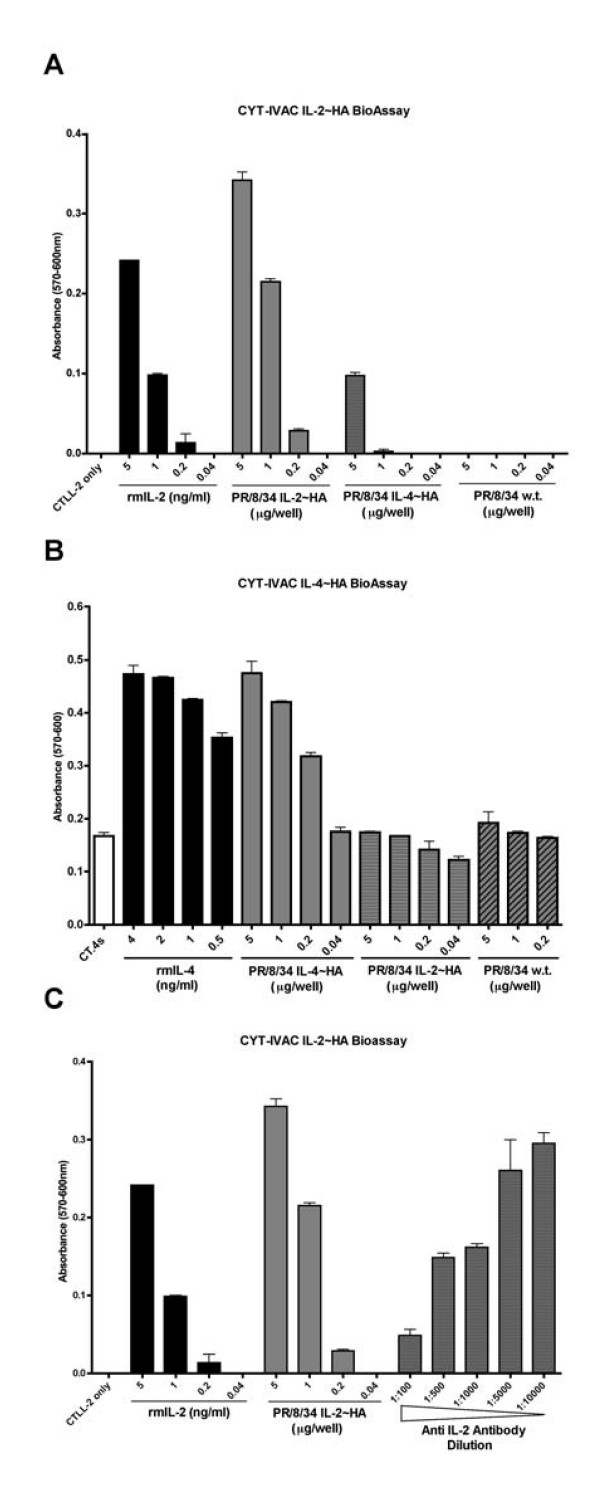
**Proliferation induced by CYT-IVACs is specific and dependent on the respective membrane-bound cytokine**. Proliferation of cytokine responsive cell lines CTLL-2 (A) and CT.4s (B) was measured following incubation with β-propiolactone inactivated mIL-2 or mIL-4 bearing CYT-IVACs. IL-2 CYT-IVAC induced proliferation of CTLL-2 cells was inhibited in a dose dependent manner with anti-mIL-2 neutralizing antibodies (C). Recombinant protein was used as a positive control.

To verify that positive bioassays were due to the presence of bioactive cytokines we included non-specific CYT-IVACs and cytokine-neutralizing antibodies in our evaluation. The IL-2 and IL-4 bioassays were shown to be specific for their respective cytokines as the IL-4 CYT-IVAC failed to induce significant proliferation of IL-2 dependent CTLL-2 cells (Figure 5A) and similarly, the IL-2 CYT-IVAC failed to induce the proliferation of IL-4 dependent CT.4s cells (Figure 5B). Furthermore, the addition of neutralizing anti-IL-2 antibodies to the culture media reduced proliferation of IL-2 CYT-IVAC stimulated CTLL-2 cells in a dose dependent manner (Figure 5C).

### CYT-IVACs enhance serum anti-viral antibodies and skew immune response toward Th_1 _mediated immunity

**Figure 6:**
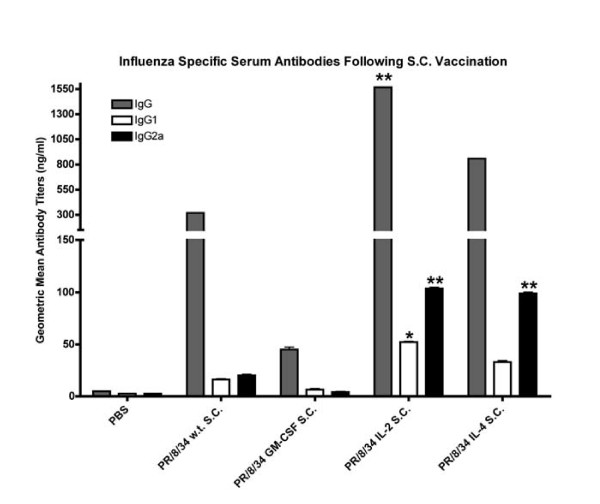
**Inactivated influenza vaccines bearing membrane-bound immunomodulators enhance serum anti-viral antibody titers**. Balb/c mice were vaccinated subcutaneously with 0.375 μg of A/PR/8/34 wild-type (n = 20) or A/PR/8/34 bearing membrane-bound GM-CSF (n = 10), IL-2 (n = 19), and IL-4 (n = 20). PBS served as negative vehicle control. Serum was collected on day 21 post-vaccination and antibody titers for influenza virus specific IgG and isotypes IgG1 (Th_2_) and IgG2a (Th_1_) were determined by ELISA. Data is displayed as the geometric mean titer in ng/ml for each group. (* p < 0.05 compared to PR/8/34 w.t., ** p < 0.01 compared to PR/8/34 w.t.)

To evaluate the adjuvant potential of our CYT-IVACs, we vaccinated groups of Balb/c mice with CYT-IVACs or wild-type vaccine administered subcutaneously (s.c.). In pilot studies, we determined the dose of inactivated, wild-type A/PR/8/34 vaccine that results in seroconversion and protection against lethal challenge in 20% of mice, the 20% mouse protective dose (MPD_20_). This dose (0.375 μg) was chosen in order to evaluate subtle immunopotentiating responses induced by our CYT-IVACs. Importantly, we chose not to include a boosting dose so that we could determine whether single dose vaccination with CYT-IVACs offered more protection than wild-type vaccine. It should also be noted that no adjuvant other than the particulate matter of the vaccine itself or the incorporated cytokine was administered. Blood was collected from mice at day 21 post-vaccination and sera were evaluated by ELISA against whole viral antigens to determine elicited anti-viral antibody titers. Following subcutaneous vaccination, significant increases in influenza specific total IgG were found in mice vaccinated with the mIL-2 bearing CYT-IVAC compared to wild-type vaccinated mice (Figure 6). While IgG levels were elevated in mice vaccinated with the mIL-4 bearing CYT-IVAC, these levels were not significantly higher that wild-type vaccinated mice. Interestingly, we found influenza specific IgG levels in mice vaccinated with the mGM-CSF bearing CYT-IVAC to be much lower than the wild-type vaccinated mice.

**Figure 7:**
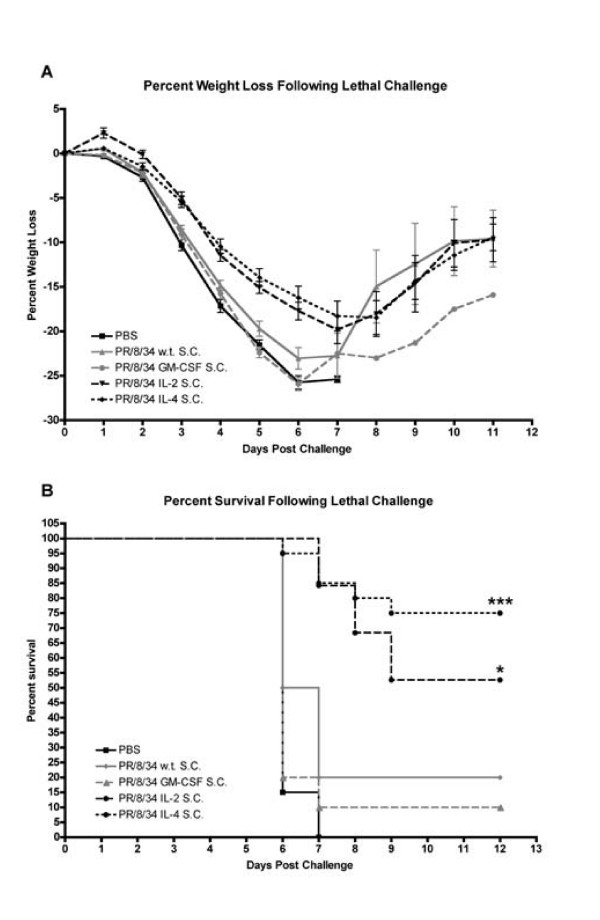
**Inactivated influenza vaccines bearing membrane-bound immunomodulators protect mice against lethal challenge**. Balb/c mice were vaccinated subcutaneously with 0.375 μg of inactivated wild-type vaccine (n = 20) or CYT-IVACs bearing membrane-bound GM-CSF (n = 10), IL-2 (n = 19), or IL-4 (n = 20) vaccine preparations. Mice were then challenged with 100 LD_50 _of mouse-adapted A/PR/8/34 on day 35 post-vaccination. PBS served as negative vehicle control. Percent weight change (A) and survival (B) were monitored over time. (* p < 0.05 compared to PR/8/34 w.t., *** p < 0.001 compared to PR/8/34 w.t.)

To further characterize the immune response elicited by CYT-IVACs we determined the influenza specific IgG_1 _and IgG_2a _levels in the serum by ELISA. It is well established that elevated IgG_1 _isotype levels, compared to IgG_2a_, is indicative of a Th_2 _mediated immune response whereas high IgG_2a _levels is indicative of a predominately Th_1_-type response. Mice vaccinated with either the mIL-2 CYT-IVAC or the mIL-4 CYT-IVAC had significantly higher IgG_2a _titers compared to wild-type vaccinated mice (Figure 7). Although significantly higher IgG_1 _titers were detected in IL-2 CYT-IVAC vaccinated mice compared to wild-type vaccinated mice, the IgG_2a _isotype remained the predominant influenza specific isotype detected in serum samples collected from mIL-2 or mIL-4 CYT-IVAC vaccinated mice, indicating a skewing towards a Th_1 _immune response.

**Figure 8:**
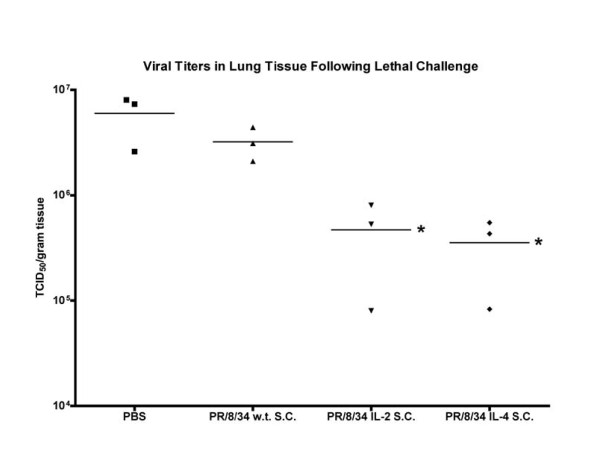
**CYT-IVAC vaccination significantly reduces viral loads in lung tissue following lethal challenge**. Mice vaccinated with either wild-type vaccine or CYT-IVACs challenged on day 35 post-vaccination with 100 LD_50 _of mouse-adapted A/PR/8/34. Mice were sacrificed on day 4 post-challenge and viral loads from homogenized lung tissue (n = 3) were determined by tissue culture infectious dose assay. Data is expressed as TCID_50 _per gram of lung tissue. (* p < 0.05 compared to PR/8/34 w.t.)

It is important to note that there was no direct correlation between elevated antibody titers and protection when evaluated on a mouse-by-mouse basis. That is, mice with high influenza specific antibody titers were not necessarily protected following lethal challenge and several mice from the IL-2 and IL-4 CYT-IVAC groups, which displayed low seroconversion titers survived lethal challenge. We were unable to detect neutralizing antibodies in any of the serum samples, however, neutralizing immune responses were clearly evoked upon challenge as viral loads were significantly reduced in the IL-2 and IL-4 CYT-IVAC vaccinated animals at day 4 post-challenge (see Figure 8). It is therefore possible that our microneutralization assay was not sensitive enough to detect the low levels of neutralizing antibody induced by the single low dose of vaccine administered.

### Vaccination with CYT-IVACs results in enhanced protection against lethal influenza virus challenge

The most compelling evidence supporting the immunostimulatory or immunomodulatory properties of our CYT-IVACs was the protection against lethal challenge. Here single dose, vaccinated mice were challenged on day 35 post vaccination with a lethal dose of mouse-adapted influenza A/PR/8/34 (100 LD_50_). Weight loss and survival were monitored following challenge. Weight loss in mice vaccinated subcutaneously with wild-type vaccine or mGM-CSF bearing CYT-IVAC closely mimicked that of PBS (sham) inoculated mice (Figure 7A). Sudden increases in percent weight loss in these groups between days 6 and 8 can be explained by a combination of recovering weight of remaining mice and loss of mice due to death; albeit mostly due to the latter. Only 20% of mice vaccinated subcutaneously with wild-type vaccine and 10% of mGM-CSF CYT-IVAC vaccinated mice were protected against lethal homotypic challenge (Figure 7B). Mice vaccinated with mIL-2 or mIL-4 bearing CYT-IVAC exhibited reduced and delayed weight loss compared to mice vaccinated with wild-type vaccine. Over 50% (p < 0.05) of mice vaccinated with mIL-2 bearing CYT-IVAC and 75% (p < 0.001) of mIL-4 CYT-IVAC vaccinated mice survived lethal challenge (Figure 7B) and those mice that succumbed to infection took considerably longer to do so.

### CYT-IVAC vaccination resulted in reduced viral loads in lungs of infected mice

In addition to evaluating protection from lethal challenge we compared viral loads in lungs of mice vaccinated with CYT-IVACs or wild-type vaccine following challenge on day 35 post vaccination. Lungs were harvested from 3 mice per vaccine group on day 4 post-challenge and viral loads of lung homogenates were determined for each mouse. We chose to omit the mGM-CSF CYT-IVAC from this study because previously recorded results indicated no adjuvant effect for this CYT-IVAC, when administered subcutaneously. Viral titers in the lungs of mice vaccinated with either the mIL-2 or mIL-4 CYT-IVAC were a full log lower compared to mice vaccinated with the wild-type vaccine (Figure 8), further confirming the enhanced protective efficacy afforded by membrane-bound cytokines on the virus particles.

## Discussion

In the present study we describe a novel approach to immunopotentiate the anti-viral, protective response induced by whole virus inactivated influenza vaccines without the need for additional adjuvants or boosting doses of vaccine. Not only were our cytokine-bearing influenza vaccines (CYT-IVACs) more efficacious than non-adjuvanted whole virus vaccine, but they skewed the elicited humoral response towards a Th_1 _mediated humoral immune response. Previously, we demonstrated feasibility of this platform for production of avian influenza vaccines bearing a membrane-bound form of chicken-derived IL-2 and GM-CSF [22]. CYT-IVAC-bearing chIL-2 significantly boosted antiviral antibody titers in vaccinated chicks compared to unadjuvanted vaccine. Here, we have extended these studies and were able to successfully develop a platform upon which membrane-bound forms of mammalian-derived immunomodulatory proteins such as mouse IL-2, IL-4, or GM-CSF can efficiently be incorporated into budding virus particles. Importantly, we confirmed that bioactivity was retained following inactivation of the virus with formaldehyde (data not shown) or β-propiolactone, two virus inactivating agents commonly used during the formulation of current influenza vaccines [32]. Further, we were able to demonstrate that the intrinsic proliferative-inducing activity associated with each individual CYT-IVACs was specific for the incorporated membrane-bound cytokine (Figure 5). This suggests that it is not simply the inclusion of the fusion protein itself that conveys immune stimulating properties, but the demonstrated bioactivity of the incorporated cytokine. It should also be noted that long-term storage (> 12 months at 4°C) did not result in any loss of cytokine specific bioactivity associated with the inactivated CYT-IVACs. In our hands, CYT-IVACs are stable and remain bioactive even following freeze/thaw when stored at -80°C.

Viral incorporation of membrane-bound cytokines is achieved through interactions between the viral matrix protein and cytoplasmic tail domains of the cytokine fusion construct, which is the same interaction used to incorporate viral hemagglutinin. Thus, there was the possibility that this platform would result in significant loss of full-length viral HA in our CYT-IVACs. Although we were unable to determine exact full-length HA protein levels, for lack of a purified standard, optical density measurements were highly similar among CYT-IVACs using HA1 (H1) specific antibodies in slot blot assays. This suggests that total HA levels were not markedly reduced in the CYT-IVACs compared to wild-type vaccine. In addition, hemagglutination units (HAU/μg total viral protein) of CYT-IVAC and wild-type vaccines did not differ significantly (Table 1). Since we did not fully understand how anchoring the cytokine to the virus particle may affect its full biological capacity, we quantitated both cytokine protein levels and specific bioactivity associated with individual CYT-IVAC formulations. There was considerable variation in the levels of incorporated cytokine based on protein content as well as associated bioactivity. For example, membrane-bound GM-CSF was incorporated at relatively high levels yet was poorly bioactive. Both IL-2 and IL-4 CYT-IVACs exhibited similar cytokine specific bioactivity, yet had variable amounts of incorporated cytokines. Of note, membrane-bound cytokine incorporation was relatively consistent across several independent vaccine preparations based on associated bioactivity per μg of viral protein (data not shown). This suggests that the observed variation in incorporation is specific for a given fusion construct and not due to variation in growth propagation of the virus in cell culture. The observed variability may partially explain why the GM-CSF CYT-IVAC, with low associated bioactive GM-CSF, did not provide better protection that the wild-type vaccine. Future formulations in which the GM-CSF molecule is extended further out from the virus particle may help enhance its bioactivity. Clearly, the amount of incorporated cytokine necessary to achieve an immunopotentiating effect will likely be cytokine specific and will require additional testing to optimize *in vivo *immunomodulatory effective dose.

Our approach of anchoring immunostimulators directly to the inactivated virus particle was designed to augment responses to current trivalent inactivated influenza vaccine platforms, which include three formulations, whole virus, split, or subunit vaccines with whole virus vaccines being the most immunogenic [33-36]. TIVs are generally well tolerated with few, if any, adverse reactions reported [37]. Adverse reactions have been reported in children vaccinated with whole virus formulations and they are generally administered split or subunit vaccines [32,38]; however, CYT-IVACs might reduce side effects of whole virus formulations if they permit the use of lower antigenic doses. Immunity induced by TIVs is dominated by humoral immunity, predominantly influenza specific serum IgG_1 _[39-42]. Our CYT-IVACs bearing IL-2 and IL-4 were both able to induce a more balanced response as evident by the higher levels of antiviral IgG_1 _and IgG_2a _antibodies compared to wild-type unadjuvanted virus vaccine. Though we did not directly assess cellular immune responses to our CYT-IVACs, isotype switching from IgG_1 _to IgG_2a _is known to be stimulated during Th_1_-type immune responses, and has been implicated in increased clearance of influenza infections following influenza vaccination [43-50]. Interestingly, the conventional immunological function of IL-4 is to stimulate Th_2 _type immune effectors and to suppress Th_1 _immunity. However, the IL-4 bearing CYT-IVAC, which induced elevated IgG_2a _antibody titers, appears to be able to polarize immune effectors in a different manner than that described for soluble IL-4 [51-54]. Other groups have reported that IL-4 in a membrane-bound form and in a highly localized environment can induce IL-12 production, a potent Th_1 _inducer, in APCs [55-58]. As noted, results obtained with the GM-CSF bearing CYT-IVAC were less conclusive and may be due in part to the reduced bioactivity of membrane-bound GM-CSF incorporated into virus particles. Large doses of GM-CSF can have an inhibitory effect on effector T cell function or lead to activation and expansion of myeloid suppressor cells [59,60]. This will require further clarification and additional studies.

Efficacy of TIVs in elderly and immunocompromised individuals is poor (30–70%) due in part to decreased immune function in these individuals that results in lower antibody titers following vaccination [32]. The inability of TIVs to effectively protect the elderly and to induce cross-protection has led to investigation of adjuvants such as Microfluidized Emulsion 59 (MF59), aluminum or toxin based adjuvants, and FLU-ISCOMs that aid in enhancing the immune response to inactivated influenza vaccines [7,8,61-69]. Our CYT-IVACs may provide the necessary adjuvant-like activity to stimulate protective responses in the elderly and this is currently being evaluated in our laboratory using an aged mouse model.

A wide range of applications exists for our cytokine-bearing viral vaccine technology. It is adaptable to a variety of species including avian, swine, canine, and equine by simply introducing species-specific immunomodulators. Likewise, human-specific immunomodulators can be incorporated in the platform for production of human specific viral vaccines. Importantly, depending on the location of the bioactive domains, immunomodulators can be presented either as type I or II membrane-bound molecules on the virus particle. This also serves to overcome potential steric hindrances that may occur during cytokine folding and/or presentation. In our laboratory, we have been able to incorporate these membrane-bound immunomodulators in H3N2, H1N1 as well as H6N2 (data not presented) influenza virus strains using the same CYT-IVAC producer cell line. Thus, vaccines against newly emerging influenza strains can be readily produced using our CYT-IVAC producer cell lines. It should also be noted, that this approach is amenable to virtually any enveloped virus, requiring only virus specific adaptation of the membrane-anchoring domain to ensure incorporation during the budding process. This approach is also amenable for inclusion of membrane-bound flagellin into baculovirus-derived influenza virus-like particles [70]. Our study provides independent evidence supporting the versatility and practicality of membrane-bound immunomodulators as effective viral vaccine adjuvants.

## Conclusion

We have demonstrated both the feasibility of viral incorporation of membrane-bound immunomodulators by influenza viruses and the enhanced efficacy of our CYT-IVACs compared to conventional, non-adjuvanted influenza virus vaccines. Superior immunogenicity of CYT-IVACs was manifested as elevated influenza specific antibodies, particularly IgG_2a _isotypes implicating Th_1 _mediated immunity. Enhanced protection from infection was also demonstrated for IL-2 and IL-4 CYT-IVAC vaccinated mice further illustrating the adjuvant effect of membrane-bound IL-2 and IL-4. The adjuvant or immune stimulating properties of CYT-IVACs makes them attractive candidates for inducing a more robust and protective immune response in the elderly and immunocompromised individuals where immune responses are waning or compromised. Further, the membrane-bound immunomodulators may be helpful in either augmenting the immunogenicity of influenza vaccines that require large antigen doses to confer protection or in reducing the dose required for protection. This could significantly increase vaccine availability targeting low immunogenic strains such as H5N1. Current studies in our lab encompassing additional immunostimulatory molecules, the intranasal route of vaccine delivery, efficacy in the aged mouse model and other enveloped virus platforms will help expand the utility and efficacy of the CYT-IVAC approach.

## Competing interests

### Patents filed

Virus vaccines comprising envelope-bound immunomodulatory proteins and methods of use thereof. Inventors: Sundick, RS, Yang, Y, Roberts, PC. US Provisional filed 7/8/2005

Virus vaccines comprising envelope-bound immunomodulatory proteins and methods of use thereof. Inventors: Sundick, RS, Yang, Y, Roberts, PC, Herbert, AS International PCT application filed July 10, 2006

## Authors' contributions

ASH was responsible for fusion construct design and assembly, establishing MDCK producer cell lines, vaccine production and characterization, completion of serological assays (ELISA, microneutralization assay), design and completion of vaccine efficacy studies, overall study design, analysis and interpretation of results, statistical analysis, drafting and reviewing the manuscript. LH participated in animal experiment design and completion. RS participated in study design and interpretation. PCR conceived the study, served as the principle investigator, participated in study design and coordination, aided in interpretation of results, helped to draft and review the manuscript.

## References

[B1] (2006). Update: influenza activity – United States and worldwide, 2005–06 season, and composition of the 2006–07 influenza vaccine. MMWR Morb Mortal Wkly Rep.

[B2] Horimoto T, Kawaoka Y (2001). Pandemic threat posed by avian influenza A viruses. Clin Microbiol Rev.

[B3] Peiris JS, Yu WC, Leung CW, Cheung CY, Ng WF, Nicholls JM, Ng TK, Chan KH, Lai ST, Lim WL, Yuen KY, Guan Y (2004). Re-emergence of fatal human influenza A subtype H5N1 disease. Lancet.

[B4] (2006). Epidemiology of WHO-confirmed human cases of avian influenza A(H5N1) infection. Wkly Epidemiol Rec.

[B5] Ungchusak K, Auewarakul P, Dowell SF, Kitphati R, Auwanit W, Puthavathana P, Uiprasertkul M, Boonnak K, Pittayawonganon C, Cox NJ, Zaki SR, Thawatsupha P, Chittaganpitch M, Khontong R, Simmerman JM, Chunsutthiwat S (2005). Probable person-to-person transmission of avian influenza A (H5N1). N Engl J Med.

[B6] Kuroda Y, Nacionales DC, Akaogi J, Reeves WH, Satoh M (2004). Autoimmunity induced by adjuvant hydrocarbon oil components of vaccine. Biomed Pharmacother.

[B7] Sambhara S, Woods S, Arpino R, Kurichh A, Tamane A, Underdown B, Klein M, Lovgren Bengtsson K, Morein B, Burt D (1998). Heterotypic protection against influenza by immunostimulating complexes is associated with the induction of cross-reactive cytotoxic T lymphocytes. J Infect Dis.

[B8] Sambhara S, Kurichh A, Miranda R, Tumpey T, Rowe T, Renshaw M, Arpino R, Tamane A, Kandil A, James O, Underdown B, Klein M, Katz J, Burt D (2001). Heterosubtypic immunity against human influenza A viruses, including recently emerged avian H5 and H9 viruses, induced by FLU-ISCOM vaccine in mice requires both cytotoxic T-lymphocyte and macrophage function. Cell Immunol.

[B9] Valensi JP, Carlson JR, Van Nest GA (1994). Systemic cytokine profiles in BALB/c mice immunized with trivalent influenza vaccine containing MF59 oil emulsion and other advanced adjuvants. J Immunol.

[B10] Leroux-Roels I, Borkowski A, Vanwolleghem T, Drame M, Clement F, Hons E, Devaster JM, Leroux-Roels G (2007). Antigen sparing and cross-reactive immunity with an adjuvanted rH5N1 prototype pandemic influenza vaccine: a randomised controlled trial. Lancet.

[B11] Kang SM, Compans RW (2003). Enhancement of mucosal immunization with virus-like particles of simian immunodeficiency virus. J Virol.

[B12] Okada E, Sasaki S, Ishii N, Aoki I, Yasuda T, Nishioka K, Fukushima J, Miyazaki J, Wahren B, Okuda K (1997). Intranasal immunization of a DNA vaccine with IL-12- and granulocyte-macrophage colony-stimulating factor (GM-CSF)-expressing plasmids in liposomes induces strong mucosal and cell-mediated immune responses against HIV-1 antigens. J Immunol.

[B13] Lee SW, Youn JW, Seong BL, Sung YC (1999). IL-6 induces long-term protective immunity against a lethal challenge of influenza virus. Vaccine.

[B14] Babai I, Samira S, Barenholz Y, Zakay-Rones Z, Kedar E (1999). A novel influenza subunit vaccine composed of liposome-encapsulated haemagglutinin/neuraminidase and IL-2 or GM-CSF. II. Induction of TH1 and TH2 responses in mice. Vaccine.

[B15] Golding B, Zaitseva M, Golding H (1994). The potential for recruiting immune responses toward type 1 or type 2 T cell help. Am J Trop Med Hyg.

[B16] Huang HI, Wu PY, Teo CY, Chen MN, Chen YC, Silin D, Tao MH (2004). Improved immunogenicity of a self tumor antigen by covalent linkage to CD40 ligand. Int J Cancer.

[B17] Oh YK, Sohn T, Park JS, Kang MJ, Choi HG, Kim JA, Kim WK, Ko JJ, Kim CK (2004). Enhanced mucosal and systemic immunogenicity of human papillomavirus-like particles encapsidating interleukin-2 gene adjuvant. Virology.

[B18] Nizard P, Gross DA, Babon A, Chenal A, Beaumelle B, Kosmatopoulos K, Gillet D (2003). Anchoring cytokines to tumor cells for the preparation of anticancer vaccines without gene transfection in mice. J Immunother.

[B19] Babai I, Samira S, Barenholz Y, Zakay-Rones Z, Kedar E (1999). A novel influenza subunit vaccine composed of liposome-encapsulated haemagglutinin/neuraminidase and IL-2 or GM-CSF. I. Vaccine characterization and efficacy studies in mice. Vaccine.

[B20] Faulkner L, Buchan G, Lockhart E, Slobbe L, Wilson M, Baird M (2001). IL-2 linked to a peptide from influenza hemagglutinin enhances T cell activation by affecting the antigen-presentation function of bone marrow-derived dendritic cells. Int Immunol.

[B21] Yei S, Bartholomew RM, Pezzoli P, Gutierrez A, Gouveia E, Bassett D, Soo Hoo W, Carlo DJ (2002). Novel membrane-bound GM-CSF vaccines for the treatment of cancer: generation and evaluation of mbGM-CSF mouse B16F10 melanoma cell vaccine. Gene Ther.

[B22] Yang Y, Leggat D, Herbert A, Roberts PC, Sundick RS (2009). A novel method to incorporate bioactive cytokines as adjuvants on the surface of virus particles. J Interferon Cytokine Res.

[B23] Donald HB, Isaacs A (1954). Counts of influenza virus particles. J Gen Microbiol.

[B24] Lawson ND, Krause DS, Berliner N (1998). Normal neutrophil differentiation and secondary granule gene expression in the EML and MPRO cell lines. Exp Hematol.

[B25] Hu-Li J, Ohara J, Watson C, Tsang W, Paul WE (1989). Derivation of a T cell line that is highly responsive to IL-4 and IL-2 (CT.4R) and of an IL-2 hyporesponsive mutant of that line (CT.4S). J Immunol.

[B26] Webster RG, Cox N, Stohr K (2002). WHO Manual on Animal Influenza Diagnosis and Surveillance.

[B27] Reed LJ, Muench H (1938). A Simple Method of Estimating Fifty Percent End Points. Am J Hyg.

[B28] Speshock JL, Doyon-Reale N, Rabah R, Neely MN, Roberts PC (2007). Filamentous influenza A virus infection predisposes mice to fatal septicemia following superinfection with Streptococcus pneumoniae serotype 3. Infect Immun.

[B29] Roberts PC, Compans RW (1998). Host cell dependence of viral morphology. Proc Natl Acad Sci USA.

[B30] Roberts PC, Lamb RA, Compans RW (1998). The M1 and M2 proteins of influenza A virus are important determinants in filamentous particle formation. Virology.

[B31] Ahmed SA, Gogal RM, Walsh JE (1994). A new rapid and simple non-radioactive assay to monitor and determine the proliferation of lymphocytes: an alternative to [3H]thymidine incorporation assay. J Immunol Methods.

[B32] Cox RJ, Brokstad KA, Ogra P (2004). Influenza virus: immunity and vaccination strategies. Comparison of the immune response to inactivated and live, attenuated influenza vaccines. Scand J Immunol.

[B33] Beyer WE, Palache AM, de Jong JC, Osterhaus AD (2002). Cold-adapted live influenza vaccine versus inactivated vaccine: systemic vaccine reactions, local and systemic antibody response, and vaccine efficacy. A meta-analysis. Vaccine.

[B34] Lund JM, Alexopoulou L, Sato A, Karow M, Adams NC, Gale NW, Iwasaki A, Flavell RA (2004). Recognition of single-stranded RNA viruses by Toll-like receptor 7. Proc Natl Acad Sci USA.

[B35] Murphy BR, Clements ML (1989). The systemic and mucosal immune response of humans to influenza A virus. Curr Top Microbiol Immunol.

[B36] Potter CW, Oxford JS (1979). Determinants of immunity to influenza infection in man. Br Med Bull.

[B37] Boyce TG, Hsu HH, Sannella EC, Coleman-Dockery SD, Baylis E, Zhu Y, Barchfeld G, DiFrancesco A, Paranandi M, Culley B, Neuzil KM, Wright PF (2000). Safety and immunogenicity of adjuvanted and unadjuvanted subunit influenza vaccines administered intranasally to healthy adults. Vaccine.

[B38] Heil F, Hemmi H, Hochrein H, Ampenberger F, Kirschning C, Akira S, Lipford G, Wagner H, Bauer S (2004). Species-specific recognition of single-stranded RNA via toll-like receptor 7 and 8. Science.

[B39] Brokstad KA, Cox RJ, Olofsson J, Jonsson R, Haaheim LR (1995). Parenteral influenza vaccination induces a rapid systemic and local immune response. J Infect Dis.

[B40] Cox RJ, Brokstad KA, Zuckerman MA, Wood JM, Haaheim LR, Oxford JS (1994). An early humoral immune response in peripheral blood following parenteral inactivated influenza vaccination. Vaccine.

[B41] El-Madhun AS, Cox RJ, Haaheim LR (1999). The effect of age and natural priming on the IgG and IgA subclass responses after parenteral influenza vaccination. J Infect Dis.

[B42] el-Madhun AS, Cox RJ, Soreide A, Olofsson J, Haaheim LR (1998). Systemic and mucosal immune responses in young children and adults after parenteral influenza vaccination. J Infect Dis.

[B43] Snapper CM, Paul WE (1987). Interferon-gamma and B cell stimulatory factor-1 reciprocally regulate Ig isotype production. Science.

[B44] Arulanandam BP, O'Toole M, Metzger DW (1999). Intranasal interleukin-12 is a powerful adjuvant for protective mucosal immunity. J Infect Dis.

[B45] Hovden AO, Cox RJ, Haaheim LR (2005). Whole influenza virus vaccine is more immunogenic than split influenza virus vaccine and induces primarily an IgG2a response in BALB/c mice. Scand J Immunol.

[B46] Huber VC, Lynch JM, Bucher DJ, Le J, Metzger DW (2001). Fc receptor-mediated phagocytosis makes a significant contribution to clearance of influenza virus infections. J Immunol.

[B47] Moran TM, Park H, Fernandez-Sesma A, Schulman JL (1999). Th2 responses to inactivated influenza virus can Be converted to Th1 responses and facilitate recovery from heterosubtypic virus infection. J Infect Dis.

[B48] Gerhard W, Mozdzanowska K, Furchner M, Washko G, Maiese K (1997). Role of the B-cell response in recovery of mice from primary influenza virus infection. Immunol Rev.

[B49] Mozdzanowska K, Furchner M, Washko G, Mozdzanowski J, Gerhard W (1997). A pulmonary influenza virus infection in SCID mice can be cured by treatment with hemagglutinin-specific antibodies that display very low virus-neutralizing activity in vitro. J Virol.

[B50] Huber VC, McKeon RM, Brackin MN, Miller LA, Keating R, Brown SA, Makarova N, Perez DR, Macdonald GH, McCullers JA (2006). Distinct contributions of vaccine-induced immunoglobulin G1 (IgG1) and IgG2a antibodies to protective immunity against influenza. Clin Vaccine Immunol.

[B51] Abbas AK, Murphy KM, Sher A (1996). Functional diversity of helper T lymphocytes. Nature.

[B52] Boothby M, Mora AL, Aronica MA, Youn J, Sheller JR, Goenka S, Stephenson L (2001). IL-4 signaling, gene transcription regulation, and the control of effector T cells. Immunol Res.

[B53] Siebenkotten G, Esser C, Wabl M, Radbruch A (1992). The murine IgG1/IgE class switch program. Eur J Immunol.

[B54] Kalinski P, Smits HH, Schuitemaker JH, Vieira PL, van Eijk M, de Jong EC, Wierenga EA, Kapsenberg ML (2000). IL-4 is a mediator of IL-12p70 induction by human Th2 cells: reversal of polarized Th2 phenotype by dendritic cells. J Immunol.

[B55] Chakrabarti R, Chang Y, Song K, Prud'homme GJ (2004). Plasmids encoding membrane-bound IL-4 or IL-12 strongly costimulate DNA vaccination against carcinoembryonic antigen (CEA). Vaccine.

[B56] Hochrein H, O'Keeffe M, Luft T, Vandenabeele S, Grumont RJ, Maraskovsky E, Shortman K (2000). Interleukin (IL)-4 is a major regulatory cytokine governing bioactive IL-12 production by mouse and human dendritic cells. J Exp Med.

[B57] Yao Y, Li W, Kaplan MH, Chang CH (2005). Interleukin (IL)-4 inhibits IL-10 to promote IL-12 production by dendritic cells. J Exp Med.

[B58] Ebner S, Ratzinger G, Krosbacher B, Schmuth M, Weiss A, Reider D, Kroczek RA, Herold M, Heufler C, Fritsch P, Romani N (2001). Production of IL-12 by human monocyte-derived dendritic cells is optimal when the stimulus is given at the onset of maturation, and is further enhanced by IL-4. J Immunol.

[B59] Parmiani G, Castelli C, Pilla L, Santinami M, Colombo MP, Rivoltini L (2007). Opposite immune functions of GM-CSF administered as vaccine adjuvant in cancer patients. Ann Oncol.

[B60] Garrity T, Pandit R, Wright MA, Benefield J, Keni S, Young MR (1997). Increased presence of CD34+ cells in the peripheral blood of head and neck cancer patients and their differentiation into dendritic cells. Int J Cancer.

[B61] Hehme N, Engelmann H, Kuenzel W, Neumeier E, Saenger R (2004). Immunogenicity of a monovalent, aluminum-adjuvanted influenza whole virus vaccine for pandemic use. Virus Res.

[B62] Ito R, Ozaki YA, Yoshikawa T, Hasegawa H, Sato Y, Suzuki Y, Inoue R, Morishima T, Kondo N, Sata T, Kurata T, Tamura S (2003). Roles of anti-hemagglutinin IgA and IgG antibodies in different sites of the respiratory tract of vaccinated mice in preventing lethal influenza pneumonia. Vaccine.

[B63] Matsuo K, Yoshikawa T, Asanuma H, Iwasaki T, Hagiwara Y, Chen Z, Kadowaki SE, Tsujimoto H, Kurata T, Tamura SI (2000). Induction of innate immunity by nasal influenza vaccine administered in combination with an adjuvant (cholera toxin). Vaccine.

[B64] Mutsch M, Zhou W, Rhodes P, Bopp M, Chen RT, Linder T, Spyr C, Steffen R (2004). Use of the inactivated intranasal influenza vaccine and the risk of Bell's palsy in Switzerland. N Engl J Med.

[B65] Squarcione S, Sgricia S, Biasio LR, Perinetti E (2003). Comparison of the reactogenicity and immunogenicity of a split and a subunit-adjuvanted influenza vaccine in elderly subjects. Vaccine.

[B66] Tamura S, Ito Y, Asanuma H, Hirabayashi Y, Suzuki Y, Nagamine T, Aizawa C, Kurata T (1992). Cross-protection against influenza virus infection afforded by trivalent inactivated vaccines inoculated intranasally with cholera toxin B subunit. J Immunol.

[B67] Tamura S, Samegai Y, Kurata H, Nagamine T, Aizawa C, Kurata T (1988). Protection against influenza virus infection by vaccine inoculated intranasally with cholera toxin B subunit. Vaccine.

[B68] Tamura S, Yamanaka A, Shimohara M, Tomita T, Komase K, Tsuda Y, Suzuki Y, Nagamine T, Kawahara K, Danbara H (1994). Synergistic action of cholera toxin B subunit (and Escherichia coli heat-labile toxin B subunit) and a trace amount of cholera whole toxin as an adjuvant for nasal influenza vaccine. Vaccine.

[B69] Tamura SI, Asanuma H, Ito Y, Hirabayashi Y, Suzuki Y, Nagamine T, Aizawa C, Kurata T, Oya A (1992). Superior cross-protective effect of nasal vaccination to subcutaneous inoculation with influenza hemagglutinin vaccine. Eur J Immunol.

[B70] Wang BZ, Quan FS, Kang SM, Bozja J, Skountzou I, Compans RW (2008). Incorporation of membrane-anchored flagellin into influenza virus-like particles enhances the breadth of immune responses. J Virol.

